# Informing the implementation of evidence-informed decision making interventions using a social network analysis perspective; a mixed-methods study

**DOI:** 10.1186/s12913-017-2067-9

**Published:** 2017-02-08

**Authors:** Reza Yousefi Nooraie, Lynne Lohfeld, Alexandra Marin, Robert Hanneman, Maureen Dobbins

**Affiliations:** 1 Institute of Health Policy, Management, and Evaluation, University of Toronto, Toronto, Canada; 2 Queen’s University Belfast, Centre for Public Health, Belfast, UK; 3 Department of Sociology, University of Toronto, Toronto, Canada; 4 Department of Sociology, College of Humanities, Arts, and Social Sciences, University of California, Riverside, USA; 5 School of Nursing and Department of Health Research Methods, Evidence and Impact, McMaster University, Hamilton, Canada; 6 175 Longwood Road South, Suite 210a, Hamilton, ON L8P 0A1 Canada

**Keywords:** Evidence-informed, Evidence-based, Social network analysis, Implementation, Mixed methods

## Abstract

**Background:**

Workforce development is an important aspect of evidence-informed decision making (EIDM) interventions. The structure of formal and informal social networks can influence, and be influenced, by the implementation of EIDM interventions.

**Methods:**

In a mixed methods study we assessed the outcomes of a targeted training intervention to promote EIDM among the staff in three public health units in Ontario, Canada. This report focuses on the qualitative phase of the study in which key staff were interviewed about the process of engagement in the intervention, communications during the intervention, and social consequences.

**Results:**

Senior managers identified staff to take part in the intervention. Engagement was a top-down process determined by the way organizational leaders promoted EIDM and the relevance of staff’s jobs to EIDM. Communication among staff participating in the workshops and ongoing progress meetings was influential in overcoming personal and normative barriers to implementing EIDM, and promoted the formation of long-lasting social connections among staff. Organization-wide presentations and meetings facilitated the recognition of expertise that the trained staff gained, including their reputation as experts according to their peers in different divisions.

**Conclusion:**

Selective training and capacity development interventions can result in forming an elite versus ordinary pattern that facilitates the recognition of in-house qualified experts while also strengthening social status inequality. The role of leadership in public health units is pivotal in championing and overseeing the implementation process. Network analysis can guide and inform the design, process, and evaluation of the EIDM training interventions.

## Background

Given the complex nature of public health systems, several factors apart from the development and provision of high-quality research influence public health decisions, such as community views, social and political pressure, and organizational constraints [1, 2]. Likewise, interpersonal, organizational and sociocultural barriers and facilitators can affect the implementation and adaptation of evidence-informed decision making (EIDM) interventions [3, 4].

Translation of research evidence into practice is a dialogic and communicative process, and health practitioners often turn to their peers as a key information source [5, 6]. A crucial aspect of EIDM in public health is development of workforce who is competent in finding and applying evidence in practice [7]. Several studies have assessed the effect of educational interventions on the knowledge and practice of health practitioners [8–10]. Individuals do not practice in vacuum and are influenced by other individuals [11] and surrounding social norms [12]. The social structure itself changes dynamically over time [13]. This inherent dependence and complexity may explain inconsistent results of studies on the effectiveness of behavior change interventions. More attention should be paid to the microstructure of inter-individual dynamics and how they influence and are influenced by the implementation of EIDM interventions.

Social network analysis (SNA) is a well-established perspective that focuses on the patterns of relationships between individuals and social groups [14, 15]. SNA examines individuals and their connecting links embedded in broader local structures beyond pair-wise relations [16], rather than treating individuals as separate units. Because of its unique perspective, SNA captures information that is generally missed by conventional survey techniques. SNA is slowly becoming more frequently used in health services research, as researchers acknowledge the complexity of health systems and the importance of networks in the translation of knowledge into policy and practice [17].

### A network analysis study

We studied the social networks of staff of three public health units in Ontario, Canada, before and after the implementation of a 22-month intervention to promote EIDM among public health professionals [18]. At baseline, three health units differed in terms of size, staffing, and commitment to EIDM. Unit A already had a 10-year strategic plan and a specific budget line to achieve EIDM, and had hired project specialists who were Masters level trained staff experienced in finding and interpreting evidence. Unit B was the largest and most geographically dispersed health unit. It identified EIDM as a strategic priority and assigned health promotion consultants to specific teams to conduct literature reviews to address practice issues. Unit C was the smallest health unit, in which responsibility for synthesizing evidence for practice rested mainly with program managers and front-line staff. The unit had dedicated some resources for capacity development.

The intervention included knowledge broker (KB) mentoring of small groups through the EIDM process to answer practice-relevant questions; one-day educational workshops; and one-to-one consultation and support by the KB on various steps of EIDM [19]. Participants in the intervention, a subset of staff in three public health units, were invited by management to join the workshops and form working groups developing evidence summaries to address local public health problems.

We previously reported on the effect of the intervention on the structure of information-seeking networks over time [20]. We used stochastic actor-oriented modeling [21] to study the longitudinal changes in social networks. We found that already known EIDM experts were more likely to be selected by management to participate in the intervention, and subsequently, information- seeking networks evolved towards a more centralized structure [20]. Additionally, individuals with higher EIDM behavior scores tended to move towards the center of networks and form clusters [20]. Central network actors who were connected to each other improved their EIDM behavior significantly, and also influenced the behavior of their peers [in press].

Although quantitative network analysis provides important insight to social structure, it may not be in-depth enough to uncover the subtle social mechanisms [22, 23]. In an explanatory qualitative study we shared the quantitative findings with selected staff and asked for their interpretation, personal experience, and contextual knowledge, aiming to understand and contextualize the quantitative findings based on the insider (emic) viewpoint of network actors. This paper reports on the results from our qualitative phase and an integration of quantitative and qualitative findings on how a network approach can inform implementations of organizational interventions.

## Methods

This is a sequential explanatory mixed-methods study [24], consisting of a quantitative assessment of the association between the network structure and implementation of EIDM, which was followed by an explanatory qualitative study. The quantitative phase includes a longitudinal analysis of the information-seeking networks and EIDM behavior of the staff before and after the intervention [20]. In the qualitative study, which was informed and guided by the findings of the quantitative phase, we provided complementary information regarding organizational processes influencing the observed patterns. The phenomenon of interest in the qualitative study was the process of information-seeking in public health units and how it interacted with an EIDM training program. The findings of the quantitative network analysis informed the criterion-based sampling [25] that continued until data saturation (informational redundancy), the point at which no new information was heard in subsequent interviews, and informational saturation, or the point at which all key findings were clearly understood by the researcher [26]. The study participants included a group of staff who were highly engaged in the intervention and participated in both baseline assessment and follow up, with the following qualifications:

- The most central staff in terms of centrality in the information-seeking network
- The staff with average centrality in the information-seeking network.

We limited the interviewees to highly engaged staff because we considered the interviewees as informants who were aware of the implementation process and could comment on different social processes happening during the implementation through both first-hand (personal experience) and second-hand experience (what they observed in the behaviour of their peers). We assumed that the informants’ experience and observations could provide a realistic picture of the process in their health units because many of the interviewees were central (popular) staff in social networks and frequently communicated with other staff regarding EIDM. However, because of the heavier involvement of participants in EIDM, it is possible that results were biased towards a more positive perspective; and we have missed some negative reactions to the intervention. In addition, the personal experience of interviewees is probably more valid than their indirect experience by observing others’ behaviour and hearing their stories [27].

We conducted focused interviews guided by an interview schedule. This ensured that all topics of interest were covered in a conversational manner allowing new questions to emerge [28]. The interview guides differed slightly across health units to reflect the quantitative findings of that unit (Appendix 1). We provided a brief summary of the quantitative network analysis of each respondent’s health unit. We asked the interviewees to comment on the effects of the intervention on the way staff interacted, on the prominence of experts in the health unit, and on the communication among the organizational divisions. The interviewees also explained how they thought the organizational structure and interpersonal communications might have influenced the success/failure of the intervention.

All interviews were audio recorded, anonymized, and transcribed verbatim with respondents’ prior permission. We stored and analyzed all the transcripts and field notes using the TAMS Analyzer software program [29]. The transcripts were analyzed using thematic framework analysis [30], by RYN who analyzed the transcripts and developed an emerging set of themes. Thematic framework analysis combines both the thoroughness of a deductive framework (based on the study’s aim, scope and propositions) and the flexibility of inductive thinking based on additional information identified in the data [31]. Analysis consisted of the following five stages: (1) *Familiarization:* immersion of the researcher in the data by repeated listening to audios, reading field notes and transcripts. (2) *Identifying a thematic framework:* discovering all key themes and concepts, by looking through the data, and referring to prior objectives and hypotheses, as well as emerging issues that rose in the study. At the end of this step, a codebook was developed, which was revised interactively during data analysis. (3) *Indexing:* applying the index to the data by annotating relevant phrases and paragraphs identifying the related themes. (4) *Charting:* rearranging the whole data based on the thematic framework, and grouping all associated parts of the text together in the form of charts. In addition to the verbatim, these charts contained more abstract summaries of respondents’ views and experiences. (5) *Mapping and interpretation:* connecting the relevant themes and redistributing them based on conceptual similarity, in order to explain and interpret the phenomena.

We presented the main themes of the qualitative analysis along with the overall interpretation of the qualitative and quantitative findings to two participants at each health unit (a total of six respondents) for their feedback (member checking). We presented quotes from transcripts in italics to distinguish participants’ views from our own. To increase clarity without changing intended meaning, where needed we removed some text segments (indicated by ‘…’) and/or added words [in square brackets]. At the end of the quoted text, we provided an alphanumeric anonymous label referring to the interviewees.

We arrayed and organized both quantitative and qualitative data in a joint-display table, as suggested by Creswell and Plano Clark [24] (data comparison: Table 2), and integrated the quantitative and qualitative findings as a coherent whole, in which the qualitative themes and patterns, along with relevant quotes to complement and expand the findings of the quantitative analysis (data integration).

## Results

We interviewed 14 individuals (five at unit ‘A’, five at unit ‘B’, and four at unit ‘C’). They were managers or unit leaders (*n* = 5), EIDM experts such as project specialists or health promotion consultants who helped staff with the EIDM process (*n* = 5), and four others (2 nurses, 1 epidemiologist, and 1 librarian). Half of the sample was central actors (fourth quartile of indegree centrality) in information-seeking and expertise networks. Six worked in a supervisory/administrative division, and the others in practice-based divisions such as family health, chronic diseases, and environmental health.

Three key themes emerged from the qualitative data were classified as: *the process of staff engagement*, *communication during trainings*, and the *relational outcomes of the implementation*. Table 1 summarized the main themes and subthemes of the qualitative analysis.

**Table 1:** The themes emerged through the qualitative analysis

Staff engagement
• *Leadership support:* the role of organizational leaders through the process of implementation
• *Relevant roles:* the relevance of staff’s formal job definitions to EIDM, and its impact on the adoption
• *Non-participatory engagement:* workload, involuntary recruitment, and ambiguity of the task as barriers of implementation
Communications during trainings
• *Communications among participants:* the effect of social support and frequency of interactions among co-participants in trainings
• *Communications with experts:* the dynamics of relationship among staff and recognized EIDM experts, such as KB, librarian, and epidemiologists
Relational outcomes
• *Recognition:* Recognition of trained staff as experts in EIDM and its effects on their social position in networks
• *The elite and the ordinary:* the selective training of a group of staff and the negative impact on the peers who were not chosen

### Staff engagement

The senior managers of each health unit invited a group of staff to participate in trainings and subsequent workgroups. At unit A, 51 staff members (8% of 638 total workforce), at unit B, thirteen staff (1% of 1068 total workforce), and at unit C, 18 (9% of 201 total workforce) participated in the intervention. Among the most central actors in information seeking and expertise networks 61% at unit A (mostly managers and project specialists), 10% at unit B (mostly health promotion consultants), and 56% at unit C (mostly program managers) participated in the intervention. Four of 5 epidemiologists at unit C who were central in information sharing networks did not engage in the intervention.

Interviewees in three units identified several factors affecting the process of engagement, which were classified into the role of *leadership support*, *relevance of staff’s roles* to EIDM, and *non-participatory engagement*:

- ***Leadership support:***

In the three health units the decision to participate in the study, the level and breadth of engagement, and the mechanism of staff recruitment in the intervention, was mainly a top-down mechanism initiated and supported by each unit’s organizational leaders and/or divisional managers.

The leaders of public health units (such as the medical officers of health-MOH) were potential initiators and champions of this process. This role was much more prominent at unit A, probably due to the charismatic character of its leaders, as explained by a project specialist: *“I think* [the leader’s] *style is to commandeer the resources that she needs. I sometimes get the impression that what* [the organization head] *wants she gets, in terms of staff time or resources or whatever.”(2-A)*

The strong message given by the units’ leaders was very effective in motivating the staff to participate, as explained by a manager at unit C: *“a message from MOH; knowing that EIDM was a priority, and I think he had sent those messages to the staff a number of times. And so all staff in the department knew, and he often would bring it up whenever he could.”(1-C)*

The role of the leader in the process of implementation was not mentioned in the interviews at unit B where decisions about study recruitment and the level of involvement was more localized at the organizational division level. Some divisions had a high participation rate and others refused to participate. As explained by a manager at unit B, *“What we had is it was really left up to different* [divisions] *to set their own level of involvement, and many of them sadly did not pick up the opportunity”(1-B).*

- ***Relevant roles:***

Program managers of health units selected the staff whose professional roles they considered relevant to EIDM. The composition of the selected group differed across health units depending on each unit’s organizational structure and how the leaders viewed EIDM in relation to staff roles. A leader at unit ‘A’ explained her selection process this way: *“We chose participants by the roles in the organization. Every specialist, every supervisor, every manager is eligible to participate. And we have systematically tried to enlist every single one.”(4-A).*

At unit ‘B’, health promotion consultants were highly represented in the selected group. However, due to their diverse backgrounds and broad definition of their roles, these consultants differed considerably in terms of their expertise in EIDM and its perceived relevance to their jobs, as explained by one consultant:“*Staff … kept saying, ’This is irrelevant to us, I have done this in my Masters’. We had such a hard time finding staff who do this kind of work; they were very resistant”. (4-B)*

The role of consultant was defined broadly at unit B, and could include roles that were not related to EIDM. However, all consultants were invited to participate in the intervention.

At unit ‘C’ nurses were the main group recruited by program managers based on their prior experience with the health problems to be addressed in evidence-based reports; as explained by a manager:

*It would be very hard to* [select trainees] *otherwise because they are assigned to specific work. So if I had a staff who was not assigned to that work, that means she has less time to do her other work… because we just don't have enough resources. (1-C).*

Staff showed resistance when they found the intervention, and EIDM in general, irrelevant to their roles and job definition, as explained by a manager at unit C:

*I found the process somewhat complicated even though I know it doesn’t necessarily meant to be, but I think the way we were seeing is that: here is the main work that we have to do and here is the process, separate, while really they should be integrated.* (4-C).

Compared to unit ‘A’, it seems that expectations from managers at units ‘B’ and ‘C’ were not as clear, in part because managers were seen as overseeing the production of evidence-based reports by nurses, rather than getting involved in their development, as explained by a manager in unit ‘C’: “[the recruitment process] *wasn't really saying all managers have to participate in the intervention.”(1-C)*

Interestingly, epidemiologists (who hold a central position in information-seeking networks) were not invited to join the intervention at unit ‘C’. One manager explained this as follows: *“ To be honest, I never thought of involving them. I thought we were supposed to keep it within our divisions.”(4-C)*. This disconnect was further reinforced by the epidemiologists’ belief that their job did not entail working with research evidence because their roles were more evaluation-oriented and *“tied to the processes” (2-C)* [indirect experience of the interviewee].

- ***Non-participatory engagement:***

At unit A, where the leaders’ involvement and interest was most prominent among the three units, the strong message by leaders asking for participation resulted in perception by some that *“staff were kind of forced to go to the workshops” (3-A).* This, in turn, negatively impacted staff’s motivation, as explained by a librarian at unit A: *“You didn’t have a choice.* [The] *organization expected you to do so. It was like force feeding the staff.”(3-A).*

A recurring theme in the interviews that appeared most frequently with participants from unit B was that at the beginning of the study the staff were not fully aware of what the intervention was about, how evidence would be useful in their practice, why they had been chosen, and what were they supposed to do with whatever they would learn in the training. As explained by a consultant: *“I kind of found we were really in the dark, I just got an email* [that said], *‘You are coming to this meeting so you are gonna look at this topic area’…and personally when this is over I don’t even know what is gonna happen.”(5-B)* Another consultant at unit B explained about the miscommunication between the managers and staff regarding the aims of the study and the reasons for choosing certain staff to participate: *“So they picked a whole bunch of health promotion consultants. So I remember a consultant* … *who wasn’t even told why she was there… It was not marketed like: ‘here is this initiative; who is interested?’ It was like: ‘you have been selected’.”(4-B)*

### Communication during trainings

After the workshop, staff were assigned to evidence-based report development teams. At unit ‘A’ the evidence review teams regularly held progress meetings moderated by the KB and the organizational leader. At units B and C, the progress meetings were more localized and limited to each work group and KB. At unit A, KB served onsite with regular office hours, but in two other units her engagement was a combination of onsite and offsite consultation. Quantitative analysis showed an increasing tendency among engaged staff to form information sharing clusters [20]. The interviewees explained about the dynamics of *communications among participants* and *communications with EIDM experts*:

- ***Communications among participants***

One result of these frequent interactions among the engaged staff was the ability to observe each other’s progress and learn from their experience, as explained by a project specialist: *“*[For] *six to eight months we were meeting every couple of weeks. So we were hearing what other people’s projects are, and watch them struggle, and think, ‘Oh! They find synthesis just as difficult* [to do as I do]’*”(2-A);* and echoed by an organizational leader: *“People often would say at the end of meeting: ’Oh! I found it so interesting that such and so were having this problem because that was my problem too.’ And there was a lot of identification with other people’s process and experience” (4-A).*

Communication among the groups was more sporadic in units ‘B’ and ‘C’, mostly limited to the separate meetings of evidence-based report teams and KB, as described by a manager at unit ‘C’: “*We had regular meetings with* [KB] *… We would meet sporadically about it; usually before meeting with* [KB]*. We didn’t have scheduled meetings. But we met* [KB] *regularly”. (4-C)*

- ***Communications with experts:***

Two key themes emerged regarding the communication of staff with EIDM experts through the implementation of the intervention: the role of the KB and the librarian.

The KB was the main deliverer of the intervention and had a critical role to fill in all steps of the process at each of the public health units, as pointed out by the organizational leader: *“Everybody in this organization sees the* [KB] *role as absolutely central for success. And every time I say, ‘Well, where I am going to get the money for this?’ I say. ‘I better find it because we are not losing it’.”(4-A)*, or by a manager at unit C: *“it was very helpful to have someone to go to, to be able to help us with those steps along the way. So I think it was that part was certainly appreciated.”* (1-C). In addition to personal competencies, the physical presence and accessibility of the KB was mentioned frequently as a reason for her popularity, as pointed out by a project specialist at unit A: *Her desk is right opposite the office of MOH. So she is not buried. She is front and center. […] Anyone who walks by can see it. (2-A)*

The KB was also widely seen as an external and neutral person, not involved in the policies and hierarchies of the department, as noted by a project specialist at unit A: *“She is objective in the sense that she is not involved in the dynamics and politics in each division, so if you go to her for advice she can provide that without having those things in mind.”(5-A)*.

Another important professional supporter of EIDM through the implementation of the intervention was the librarian associated with each unit. Although the public health librarians were considered to be an integral part of EIDM process by the informants in all the three health units, the perceived level of involvement and usefulness of librarians differed considerably across sites. At unit A, during the study period the unit hired new librarians who were formally assigned to do rapid reviews and develop and update search strategies. Likewise at unit C the librarian was involved in the process and was helpful in assisting staff through EIDM steps. In contrast at unit B a recurring theme in interviews was that the library system did not help staff meet EIDM standards. The library basically provided *“a million single studies”* and the staff struggled with classifying and appraising the information, as expressed by a consultant at unit B:

*One of my biggest frustrations, … is* [the study] *was trying to work organizationally with* [Unit B]*, and one of the greatest barriers is the way our library access is used… When you request a search by the library you get a stack of papers with no order, a mix of single studies and systematic reviews. You get a hodge-podge which for most of us… I wouldn’t have before known how to tease* [out] *what was what, how to quickly go through and see which one was synthesis and which one weren’t. It is a bit overwhelming. (2-B)*

### Relational outcomes

Especially at units A and C, completed reviews were presented in department-wide research events and other local meetings. Quantitative analysis [20] showed that the information seeking networks evolved towards a more centralized structure over time, in which the staff who were already central at baseline, staff with higher baseline EIDM behavior scores, and larger improvement in their EIDM behavior scores gained even more centrality. Only at unit A highly engaged staff also shifted towards the center of information seeking networks. Interviewees mostly focused on the *recognition* of participants in trainings:

- ***Recognition:***

Especially at units ‘A’ and ‘C’, trained staff had various opportunities to present their work to a larger audience both inside and outside of the health unit. As one manager noted, *“Twice a year we have our research and knowledge exchange symposium, and so all the unit is there to hear about EIDM. And they see and hear from various sources who is knowledgeable on the topic” (1-C).* Presenting work in those venues resulted in widespread recognition of trained staff by their peers, as expressed by a leader at unit ‘A’: *“If your work has been showcased in that venue, people from all over would say, ‘Oh, you did a really good job on that’. They might not even know the name of that person before, and all of a sudden they know who they are.”(4-A)*

Organizational leaders played a significant role in recognizing trained staff, as described by a manager at unit ‘A’: *“Because there is kind of a little bit of mystique about* [this] *rapid review business* … [the unit leader] *makes a big fuss about it. When you get in the meeting with* [the leader], *there is a fair amount of social capital attached to joining the rapid review* [team]*. It’s a little bit like you are kind of famous!”(1-A)* Recognition of the newly gained expertise of trained staff also occurred through word of mouth, as pointed out by a manager at unit ‘C’: *“Here we are a smaller division; lots of people just knew* [who were engaged in the intervention] *by osmosis. Because we talk* [with each other] *at our managers’ meetings.”(4-C)*

In contrast, in the larger and more diffuse public health unit ‘B’, word of mouth was not as frequently effective in promoting recognition of trained staff : *“The people who were involved were selected and were sent stuff electronically;* [but] *that wasn’t in our newsletter or anything. So I don’t think they had any exposure.”(4-B)*

- ***The Elite and the Ordinary:***

An interesting and unanticipated consequence of unit A’s strategy to target project specialists and managers, and promote the individuals who were engaged in EIDM activities was a negative reaction of the staff who were not chosen to take part in the intervention. The selected staff enjoyed working in an *“ivory tower”* environment of recognition and prestige. But many staff who were not chosen felt left behind, as indicated by a librarian at unit A*: “That mechanism of picking resulted in emotional responses for not being chosen… because they were not viewed as elite. They were not part of the club.”(3-A)* [indirect experience], As another project specialist at unit A noted [indirect experience]:

*I think the front line staff that were not been sent to* [the university-affiliated one-week workshop]*, they felt left behind and frustrated, because it was like all these staff specialists are moving forward and advancing their skills, and they are gonna be used more and appreciated more by management, again this is the sense I got. That definitely caused tension, feeling of that ivory tower of the specialists. (5-A)*

Paradoxically, being chosen for training resulted in a heavier workload and more pressure due to greater responsibilities. Prestige and workload were positively correlated, as explained by a project specialist: *“but there is also more pressure on us too, so it goes both ways. For if you got more trained there is also more pressure on you to do more work” (5-A);* or pointed out by a leader at unit A: *“We happen to have created very hard work that seen as very desirable to do.”(4-A)*

The informants at units B and C did not observe such reactions among staff. For example, when asked about the possibility of such consequences a public health nurse at unit C indicated: “*it is not about the prestige*” (3-C). The staff who were engaged only became more skilled to help others and not necessarily more popular or advantaged.

## Discussion

The results from the quantitative and qualitative phases of this mixed-methods study were integrated into a framework of how network analysis can inform the implementation of EIDM training interventions (Table 2), and is explained further below.

**Table 2:** Integration of quantitative and qualitative findings of a network analysis perspective to the implementation of EIDM in public health organizations

Quantitative highlights	Qualitative themes	Integration
Engagement in EIDM training		
• Health units A and C had higher engagement rate (8% of the staff in unit A, 1% in unit B, and 9% in units C). • The engagement rate of central actors in unit A, B, C was 61%, 10%, and 56% respectively. • At unit A, most of the engaged staff were managers and project specialists. • At health unit B, most of the engaged staff were health promotion consultants, most of whom were not central actors. • At health unit C half of the central actors were epidemiologists who mostly did not engage in the intervention. • Central network actors had higher baseline EIDM behavior scores than others.	• Organizational leaders at units A and C strongly promoted the intervention • Especially at unit A, the leaders actively monitored the progress, and controlled the quality of the output • The main mechanism of choosing staff to participate in trainings was the relevance of their roles to EIDM and the health problem. • The staff generally did not have given much choice at time of recruitment, and were not optimally informed about the value of the study and the importance and consequences of their participation. • The relevance of health promotion consultants’ role to EIDM at unit B was not clear for some staff, which resulted in negative reactions. • Epidemiologists at unit C did not engage in the intervention because they were not assigned to programs, and did not believe EIDM was relevant	• *Leadership support:* *Staff are more likely to adopt EIDM if organizational leaders strongly support it and directly engage in the process* • *Positional compatibility:* *Staff are more likely to adopt EIDM if its relevance to their formal roles is clear* • *Participatory engagement:* *Staff are more likely to adopt EIDM if they are clearly informed about the training processes and expectations, and feel in control over participation*
Networking and communication		
• Only at unit A, the KB was identified as a central staff. Even though she was not a formal employee • In three health units highly engaged staff showed a tendency to form clusters.	• KB was the main deliverer of the intervention. (Especially at unit A) • Librarians, if get engaged, supported the EIDM process • Co-participation in workshops and working on the same evidence summary provided the staff with an opportunity to share their concerns and progress with their peers and shape new social ties, if they were sustained by regular communications (progress meetings)	• *Support networks:* *Sharing experiences and concerns in regularly scheduled meetings of EIDM trainees facilitate the development of an atmosphere of trust among engaged staff.* • *EIDM champions:* *the KB and librarian are main motivators and deliverers of EIDM training and support. Their professional competence, social engagement, and physical accessibility affect implementation success*
Recognition		
• At unit A, highly engaged staff became more popular • Staff with higher baseline and higher improvement in EIDM behavior scores became more popular • Network became more centralized around already central staff	• Some of the highly engaged staff became widely popular after presenting their findings in department-wise events, being promoted by the leaders, and word of mouth • At unit A (where engagement in the intervention resulted in a considerable prestige effect) the staff who were not chosen responded negatively to the unequal carrier promotion opportunities and the ‘ivory tower’ position of project specialists	• *Recognition and promotion*: *Trained staff become more central in networks if they have the opportunity to be recognized as experts in EIDM through presentations at organization-wide events and endorsement by organizational leaders.* • *Positional advantage:* *The positional advantage of central network actors through the selective training interventions results in a “rich get richer” pattern. Selective training, on the other hand, may result in negative reactions by the staff who were not chosen.*

### Engagement in EIDM training

Network analysis can provide insight into the contextual barriers and facilitators of implementation [32]. Four themes were identified in this mixed methods study to inform the engagement process (Table 2): *leadership support*, *positional compatibility*, and *participatory engagement*.

#### Leadership support

Leadership can use their power to promote and support the implementation process. Leadership support is considered to be an important facilitator of the implementation of EIDM in health organizations. The role of leaders in promoting EIDM extends beyond inducing and prescribing EIDM behavior. Stetler et al. in a qualitative study of the role of leadership in developing, enhancing, and sustaining EIDM as the norm in health organizations, found that in addition to the ability to ‘inspire and induce’ EIDM activities, leaders intervened actively and were involved directly in EIDM activities [33]. In our study, the leaders helped staff learn about EIDM “how to’s” by becoming engaged in education and development, role modeling and monitoring the adoption process. This highlights the key role of organizational leaders who, as the champions, initiators, and role models of change interventions [34] should stimulate and monitor the implementation process.

#### Positional compatibility

Health units differed in terms of the compatibility between formal roles and network positions. Implementation models suggest that effective programs require four key individuals: *champions*, *opinion leaders, formally appointed internal implementation leaders,* and *external change agents* [35]. At unit A, a group of project specialists were hired and trained to lead EIDM in the unit (*formally appointed internal implementation leaders*) and were already central in information-seeking networks due to their professional activities (*opinion leaders*), and were among the first groups engaged in the intervention (*champions*) [36, 37]. The overlap between these roles was less prominent in the two other health units. For example, the epidemiologists at unit C and some health promotion consultants at unit B who were already central in the network at baseline [20], did not engage in the intervention because they did not consider EIDM relevant to their jobs. Managers also felt differently about their role in the EIDM process, and considered themselves as overseers, and not direct players in the process. These highlight the need to recognize the compatibility between various social and organizational roles of health practitioners as a factor determining the adoption of innovations. The interventions should be compatible with the values, needs, and perceived risks of involved individuals [38, 39]. EIDM is relevant to a broad range of organizational roles from front-line staff to senior managers, and a single skill set in EIDM does not reflect the diversity of public health roles [40]. Instead of a generic educational package, the training program should have been customized to different key players, for example, focusing on technical aspects for professional groups and supervising and role modeling aspects for managers [41].

#### Participatory engagement

Another insight from the qualitative analysis regarding the factors affecting the engagement in the intervention was that the engagement process was generally a top-down, non-voluntary mechanism. Some informants noted the negative reactions and resistance of some staff to the intervention. Their resistance was mainly due to the involuntary nature of staff recruitment (staff generally were not given much choice and were not optimally informed at the time of recruitment in the study), added workload and high expectations by leaders, a perception of incoherence between EIDM and the norm of public health practice, and a perceived disconnect between EIDM training and real public health problems. Greengalgh et al. in a systematic review of models of innovation diffusion, highlighted the importance of compatibility of an intervention with the values, needs, and perceived risks of involved individuals [38]. Interventions that are not considered to be in line with professional and organizational values, missions and competencies face resistance by health practitioners [39]. Providing staff with knowledge regarding the relative advantage of a new innovation, its compatibility with current values and norms, and adaptability of the innovation to the needs of potential adopters are a few of the factors that support the adoption of innovations [38]. This also highlights the importance of collaborative networking, decentralization of decision-making, and provision of a safe environment, as important strategies helping the organizational leaders implementing EIDM in health organizations [42, 43].

### Networking and communication

Network analysis can also inform the design and delivery of training interventions [44, 45]. Two main themes in our study were the formation of *support networks* among engaged staff, and communications with the *EIDM champions* (Table 2).

#### Support networks

During the trainings, the communication among the participants provided a safe context for information sharing and feedback. The tendency of engaged staff to form clusters consisting of individuals who have similar expertise and interests and can help each other through communication and feedback implies the formation of communities of practice [46, 47]. These communities provide a safe and non-judgmental context that supports information sharing and feedback [48, 49]. If continued, the members of the communities of practice develop tacit knowledge and a repertoire of solutions to shared problems that facilitate the spread of knowledge and access to professional help in the long term, and increases the productivity of the system [46, 50]. However, the formation of cohesive clusters should also coincide with the formation of bridging connections to the periphery to minimize the entrapment of knowledge in silos [51]. Program implementers can facilitate the formation of communities of practice by providing regular and sustainable networking opportunities to the staff from different teams, in the form of progress and support meetings.

#### EIDM champions

Developing and maintaining inter-personal and inter-unit networks are considered as one of the main activities of KBs [41]. KB’s personal competencies and professional skills, her physical presence and accessibility, her recognition and support by organizational leaders, and her objectivity and independence from local politics of health units were among the main reasons for the essential role of KB in the process of change and her central position in social networks, as explained by the interviewees.

In this study, librarians at units A and C also acted as objective, independent, and knowledgeable information sources for staff of various divisions. This bridging role is consistent with what some scholars suggested for librarians, to be seen as more than mere suppliers of the information and to communicate with and connect various disciplines and groups [52]. The advantageous position of librarians in social networks could partly be explained by their bridging role in connecting separate segments of the network and providing access to non-redundant information about other groups [53].

### Recognition

Two main themes of the analysis of network outcomes of the implementation were the *recognition and promotion* of trained staff and also the *positional advantage* of already central network actors (Table 2).

#### Recognition and promotion

Presentation of evidence reviews in organization-wide conferences and other events facilitated recognition of the trained staff as EIDM experts (especially at unit A). Increased centrality of the trained staff is a favorable outcome for a training intervention aiming to empower a selected group of individuals; implying that their peers recognized their expertise and turn to them for information. The majority of public health workers lack formal training and expertise in EIDM [54, 55]. So the existence of accessible local experts facilitates the process of EIDM in public health organizations. In order to achieve that goal, in addition to training, recognition and promotion channels should be developed, through which the trained experts be added to the referral directory of more people in the organization [56].

#### Positional advantage

The intervention in this study included EIDM training of practitioners selected by unit managers. The managers’ choices were often based on identifying staff whose work was already most closely tied to EIDM. Therefore being an already central network actor increased the chance of being selected. Central network actors have access to more resources and are more likely to be aware of promotional opportunities in organizations [57]. In addition, because of their favorable social position they are more likely to engage in risky behaviors and new innovations [58], which is a necessary characteristic of early adopters [59]. The potential to influence others, and their tendency to try innovations make the central network actors suitable individuals to engage in organizational interventions [60, 61]. We recommend considering the engagement of central network actors in interventions that would benefit from peer influence by local opinion leaders [60, 62].

Already central network actors became even more central after intervention [20]. The positional advantage of central experts and subsequent presentation at events, and promotion by the leaders resulted in a preferential increase in their centrality, leading to the “rich get richer” phenomenon [63, 64], which may lead to better access to high quality resources [56]. Increased centrality coincides with an increase in social power and ability to influence the behavior of others and to promote innovations [65]. However, deep inequality in social status may act as a barrier to communication [66], and decrease the availability to help when needed [67].

## Conclusions

In conclusion, social network analysis can be used to inform various stages of the implementation. It informs the engagement process by considering the social position of staff as a selection factor. A network approach to training interventions could facilitate communication and formation of support communities. A network perspective can also inform the evaluation of implementation success, by assessing the changes in the social position of participants and their subsequent social dynamics as a contributing factor in the sustainability of implementation.

The analysis of social networks comes with its own challenges that the researchers should be aware of and prepared for. Extra efforts should be made to reduce harm to participants and preserve their confidentiality [68]. Some network indicators are more sensitive to non-response, especially when the reason for missing values is not due to random error [69, 70]. We suggest balancing the decision to run a network analysis as a part of an implementation strategy with considerations regarding the design and administration challenges, and preferably complementing a quantitative SNA with a qualitative analysis of the perspectives and experiences of network actors.

## Appendix 1 qualitative interview guide

*(The interviewer thanks the interviewee for participation. He explains that the interview (s) will be coded and that the personal information being collected from participants (i.e., name, position, work address, telephone number, email) as well as the code list will be kept separately from the interview.*

*The interviewer continues with an introduction to the study objectives and methods:*

- A longitudinal analysis to assess how a tailored KT intervention affected the pattern of knowledge flow, the distribution of power in the organization, and the development of interdivisional partnerships.
- Comparing the longitudinal changes in networks in different public health departments with different contextual and organizational characteristics, with the aim of understanding the role of context and organizational culture on the implementation process.
- Following the longitudinal analysis, the qualitative study will assist in translating the quantitative SNA findings into the real life experience of the staff, helping us understand how staff envision their position in the social network, and how they interpret the observed changes in the network shape over time, as an insider.
- Together the quantitative and qualitative analysis will inform the development of future KT interventions and will expand our knowledge about the mechanisms of KT in health care systems.

*Then the interviewer reviews the process of network surveys and the four network questions that the respondents answered in online surveys. He explains that the lists provided by each respondent were combined and transformed into actor by actor matrices in which each cell represents whether actor A sought information from actor B, recognized actor B as an expert, and identified her as her friend. In those matrices some actors identified by more peers as information sources or experts. This determined the centrality of actors in the networks. Statistical techniques were used to model the formation that centrality and its changes through time.)*

- **Please think about the recent occasions that the staff asked you helping them inform their decisions using research evidence.**

- ○ What kind of help did the staff ask from you?
- ○ What factors have led to you being identified as an information source?

*Probes:*

*Expertise, personal characteristics, formal job definition, frequency of interaction, availability, informal connections*

- How have you influenced the way the staff use research evidence in practice?

*Probes:*

- *Verbal influence*
- *Non-verbal influence*

- **Please think about the recent occasions that you asked a peer in the health unit for help informing your decisions using research evidence.**

- ○ What kind of help did you ask for from these staff?
- ○ What qualifications do you consider for a person to turn to for getting help in issues relevant to finding and using research evidence?

*Probes:*

*Expertise and knowledge, job title/professional role, similarity of characteristics, ease of access and frequency of contacts, friendship*

- ○ How your interactions with other staff have influenced shaping of your current behavior and attitudes on using evidence in practice?

*Probes:*

- *O bserving their behavior in daily practice*
- *Being influenced by their expertise and professionalism*
- *Informal chatting about those issues*
- *The role of friendship and trust*

*The researcher will explain briefly about the main findings of the quantitative analysis. For each piece of findings seeks the opinion of the interviewee, her experience regarding those finding, and possible confirming/disconfirming examples:*

- ***Unit A:***

- There were a group of staff in the department who were more central than others in information seeking and expertise networks (more people turned to them asking for information, and recognized them as experts). We call them the local opinion leaders.
- Most of these local opinion leaders were from office of the medical officer of health and family health divisions.
- A large percentage of these local opinion leaders were intensively involved in KT intervention.
- The majority of these local opinion leaders who were intensively involved were managers and project specialists.
- In average these opinion leaders who were intensively involved in KT were also more central than others in friendship network (had larger friendship networks than average staff).
- These opinion leaders who were intensively involved in KT already had significantly higher scores of evidence-based practice behavior at baseline, compared to the rest of staff.
- After intervention, the staff who were intensively involved in KT, turned to each other for seeking information in a more reciprocal way than before (if Jack turned to Joe, Joe also turned to Jack asking for information). But that increase did not happen in the staff who were not intensively involved.
- Likewise, the staff who were intensively involved recognized each other as experts in a more reciprocal way at follow up, compared to baseline.
- A group of intensively involved opinion leaders who sought information from each other significantly improved their EBP behavior scores over time. But that improvement did not happen in other staff who were intensively involved in KT but were not opinion leaders.
- The staff who sought information from those opinion leaders also showed a significant improvement in EBP behavior scores over time, regardless of their involvement.
- The improvement in EBP behavior was associated with shifting one’s position towards the center of the network/being identified by more people as an information source. In other words, the more people improved their EBP behavior, the more they identified by peers as information source (or vice versa)
- Averagely, the staff who were intensively involved in KT significantly improved their position in the information-seeking network. Even though it did not necessarily coincide with improvement in their EBP behavior.
- Many staff identified the knowledge broker as an information source and expert. Even though the knowledge broker was not an official staff in the department. This did not happen in any of other 2 health departments in the study.

- ***Unit B:***

- the number of staff who were intensively involved in KT was the lowest among the three study departments, even though department C was the largest department in the study.
- There were a group of staff in the department who were more central than others in information seeking and expertise networks (more people turned to them asking for information, and recognized them as experts). We call them the local opinion leaders.
- Most of these local opinion leaders were from Chronic disease prevention and performance and standards directorates.
- Only a very small fraction of these local opinion leaders were intensively involved in KT intervention.
- After intervention, the staff who were intensively involved in KT, turned to each other for seeking information in a more reciprocal way than before (if Jack turned to Joe, Joe also turned to Jack asking for information). They also recognized each other as experts in a more reciprocal way than before.
- The improvement in EBP behavior was associated with shifting one’s position towards the center of the network/being identified by more people as an information source In other words, the more people improved their EBP behavior, the more they identified by peers as information source (or vice versa).
- Averagely, the staff who were intensively involved in KT significantly improved their position in the information-seeking network. Even though it did not necessarily coincide with improvement in their EBP behavior.

- ***Unit C:***

- There were a small group of staff in the department who were more central than others in information seeking and expertise networks (more people turned to them asking for information, and recognized them as experts). We call them the local opinion leaders.
- Most of these local opinion leaders were epidemiologists from Administration division, and managers in public nursing and nutrition.
- Half of these local opinion leaders were intensively involved in KT intervention.
- The majority of these local opinion leaders who were intensively involved were managers and directors.
- A group of intensively involved opinion leaders who sought information from each other significantly improved their EBP behavior scores over time. But that improvement did not happen in other staff who were intensively involved in KT but were not opinion leaders.
- The staff who sought information from those opinion leaders also showed a significant improvement in EBP behavior scores over time, regardless of their involvement.
- The improvement in EBP behavior was associated with shifting one’s position towards the center of the network/being identified by more people as an information source. In other words, the more people improved their EBP behavior, the more they identified by peers as information source (or vice versa)
- The improvement in EBP behavior was associated with shifting one’s position towards the center of the network/being identified by more people as an information source. In other words, the more people improved their EBP behavior, the more they identified by peers as information source (or vice versa)

- **In what ways, do you think, the KT intervention affected the way staff turn to each other seeking information?**

- ○ How did it influence how staff get help from each other?
- ○ How did it influence how staff get help from you?
- ○ How did it influence your own interaction with other staff?
- ○ How did it affect communication among the divisions?

*Probes:*

- *The frequency of interactions*
- *The types of help needed*

- *Change in the definition of expertise*
- *Identification of new experts*
- *Staff autonomy and independence*
- *Formation of new clusters in the network*

- ○ How did the pattern of communication among the staff in the health unit affect the implementation of KT intervention?
- ○ How did the organizational structure of the health unit affect the implementation of KT intervention?

*Probes:*

- *The role of formal job definitions*
- *The role of informal, friendly communications*
- *Accessibility*

What do you think we might have missed or have shown incorrectly in the analysis?

## Abbreviations

EIDM: Evidence-informed decision making
KB: Knowledge broker
MOH: Medical officer of health
